# Factors Moderating the Association between Cannabis Use and Psychosis Risk: A Systematic Review

**DOI:** 10.3390/brainsci10020097

**Published:** 2020-02-12

**Authors:** Sanne J. van der Steur, Albert Batalla, Matthijs G. Bossong

**Affiliations:** Department of Psychiatry, University Medical Center Utrecht Brain Center, Utrecht University, 3584CX Utrecht, The Netherlands

**Keywords:** cannabis use, psychotic disorder, genetics, age of onset, clinical high risk

## Abstract

Increasing evidence indicates a relationship between cannabis use and psychosis risk. Specific factors, such as determinants of cannabis use or the genetic profile of cannabis users, appear to moderate this association. The present systematic review presents a detailed and up-to-date literature overview on factors that influence the relationship between cannabis use and psychosis risk. A systematic search was performed according to the PRISMA guidelines in MEDLINE and Embase, and 56 studies were included. The results show that, in particular, frequent cannabis use, especially daily use, and the consumption of high-potency cannabis are associated with a higher risk of developing psychosis. Moreover, several genotypes moderate the impact of cannabis use on psychosis risk, particularly those involved in the dopamine function, such as AKT1. Finally, cannabis use is associated with an earlier psychosis onset and increased risk of transition in individuals at a clinical high risk of psychosis. These findings indicate that changing cannabis use behavior could be a harm reduction strategy employed to lower the risk of developing psychosis. Future research should aim to further develop specific biomarkers and genetic profiles for psychosis, thereby contributing to the identification of individuals at the highest risk of developing a psychotic disorder.

## 1. Introduction

Schizophrenia and other psychotic disorders are a burdensome group of disorders, with occurrence of psychosis as an overlapping phenomenon and a lifetime morbid risk of about 7 per 1000 [1,2]. Characteristics of psychosis include positive symptoms, such as hallucinations and delusions; negative and affective symptoms, such as a lack of motivation and depression; and neurocognitive alterations [2]. Although one of the most robust pathophysiological features of psychosis is an increase in the striatal dopamine function, accumulating evidence indicates abnormalities in the endocannabinoid system of patients with a psychotic disorder [3,4,5]. For example, patients exhibit enhanced levels of endogenous cannabinoid ligands in cerebrospinal fluid [6,7], as well as increased post-mortem CB1 receptor densities [8,9] and in vivo CB1 receptor availability in the brain [10,11]. 

A convincing amount of evidence indicates an association between cannabis use and the risk of psychosis. First, the administration of Δ9-tetrahydrocannabinol (THC), which is the main psychoactive component of cannabis, to healthy individuals can induce transient psychotic-like experiences [12,13,14] (see for a review, [15]). In addition, early epidemiological studies showed a relationship between cannabis use and the development of psychotic symptoms or a psychotic disorder later in life [16,17,18,19,20,21,22]. Systematic reviews and meta-analyses of these data have confirmed cannabis use as a risk factor in the development of psychosis or schizophrenia [23,24,25,26]. In particular, Moore et al. concluded that the risk of any psychotic outcome in individuals who had ever used cannabis was increased by approximately 1.5 times [26]. 

Specific factors appear to moderate this association between cannabis use and the development of psychosis, in particular, determinants of cannabis use. Several studies have shown that a higher frequency of cannabis use is related to a higher risk of psychosis [18,19] (see for reviews and meta-analysis, [26,27,28]). Similarly, a lower age of onset of cannabis use [17,19,21] and the use of more potent types of cannabis, with higher THC and lower cannabidiol (CBD) concentrations [22,28], have been shown to further increase the risk of psychosis. CBD is a non-intoxicating cannabinoid compound that may attenuate some of the negative effects associated with cannabis use [29,30,31]. The genetic profile of cannabis users has also been implicated as a moderator of the association between cannabis use and the development of psychosis [32,33,34]. For example, the catechol-O-methyltransferase (COMT) Val158Met genotype, involved in dopamine turnover in the prefrontal cortex, was shown to interact with the impact of cannabis use on psychosis risk [35,36]. Initial studies also suggested that cannabis use is associated with an earlier age of psychosis onset [37,38,39,40]. Finally, cannabis use by individuals at a clinical high risk (CHR) for psychosis may exacerbate psychotic symptoms and drive an earlier transition to psychosis [41], although contrasting results have also been reported [42].

The present systematic review aims to give an up-to-date, detailed overview of the most recent literature (2009–2019) on factors that influence the relationship between recreational cannabis use and the risk of psychosis. The reviewed articles investigated how this relationship was affected by (1) patterns of cannabis use (e.g., dose and frequency); (2) age of initiation of cannabis use; (3) type of cannabis used; and (4) the individual genetic profile. Studies on the age of onset of psychosis and the influence of cannabis use on the transition to psychosis in people at CHR are also discussed.

## 2. Materials and Methods

### 2.1. Literature Search and Selection Procedures

A systematic search was performed in two databases—MEDLINE and Embase—to identify relevant studies conforming to the Preferred Reporting Items for Systematic Reviews and Meta-Analyses (PRISMA) guidelines [43]. The final systematic search was performed on July 23rd, 2019. The search was limited to human studies published between 2009 and 2019 and was run through titles and abstracts. The exact search term used was as follows: 

((((("Cannabis"[Mesh]) OR ((Cannabis[Title/Abstract] OR Marihuana*[Title/Abstract] OR Marijuana*[Title/Abstract] OR Hashish*[Title/Abstract] OR Hemp[Title/Abstract])))) AND (("Psychotic Disorders"[Mesh]) OR ((psychotic disorder*[Title/Abstract] OR psychosis[Title/Abstract] OR psychoses[Title/Abstract] OR psychotic[Title/Abstract]))))) NOT (animals[MeSH Terms] NOT humans[MeSH Terms]).

**Figure 1:**
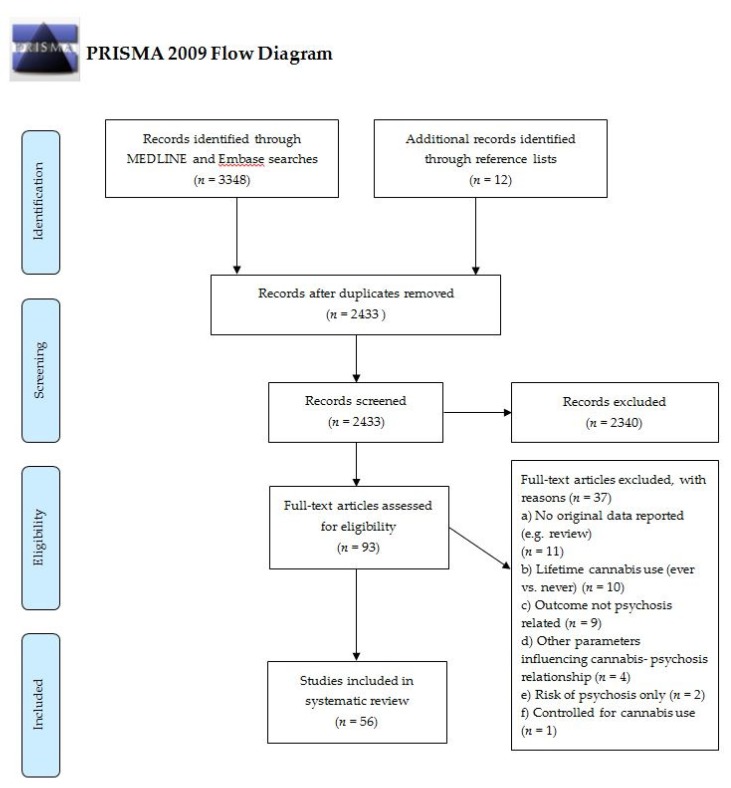
Literature search and selection of studies adapted from the Preferred Reporting Items for Systematic Reviews and Meta-Analyses (PRISMA) flowchart (http://www.prisma-statement.org/).

Further publications were found by screening reference lists from included articles and relevant reviews. The PRISMA flowchart presented in Figure 1 shows the selection procedure employed to identify relevant studies. A total of 3348 records were identified through the search. An additional 12 records were found through screening reference lists of relevant articles. After the removal of duplicates, 2433 articles were screened and 2340 records were excluded. In total, 93 records were assessed for eligibility, leaving 56 studies to be included. Please see Table S1 in the Supplementary Materials for an overview of included studies.

### 2.2. Selection Criteria

Publications were screened by two researchers. Only published, peer-reviewed, and observational studies investigating the relationship between cannabis use and psychosis were considered and were selected when they examined one of the following moderating factors: (1) patterns of cannabis use (e.g., dose and frequency); (2) age of initiation of cannabis use; (3) type of cannabis used; (4) the individual genetic profile; (5) cannabis use related to the age of onset of psychosis; and (6) the influence of cannabis use on the transition to psychosis in individuals at CHR. 

If cannabis was not the only substance being investigated, studies were included only if cannabis was the most frequently used illicit substance, when each analysis was conducted separately for each substance, or when other substance use was controlled for. Studies that exclusively reported measures of lifetime cannabis use (ever vs. never), that only examined other potential risk factors for psychosis (e.g., childhood trauma), or that reported data from overlapping cohorts were excluded.

### 2.3. Statistical Outcome Measures 

Because the included papers analysed and reported their results in different ways, an explanation of statistical outcome measures is provided in Table 1. 

## 3. Results

### 3.1. Cannabis Use and the Development of Psychotic Disorders 

#### 3.1.1. Patterns of Cannabis Use

In the last decade, six studies have been published that have examined the role of patterns of cannabis use in the incidence of psychosis. In their study issued in 2009, Di Forti and colleagues found that first-episode psychosis (FEP) patients were more likely to be current cannabis users (odds ratio (OR) = 6.4, 95% Confidence Interval (CI) 3.2–28.6, *P* < 0.05) and to have used cannabis for over 5 years (OR = 2.1, 95% CI 0.9–8.4, *P* < 0.05) compared to a healthy control group [22]. In an international follow-up study published in 2019, Di Forti et al. took six measures of cannabis use across eleven European sites in more than 900 FEP patients and 1200 controls. Daily cannabis use increased the odds of psychotic disorder to 3.2 (fully adjusted OR, 95% CI 2.2–4.1, P < 0.0001) compared with never having used cannabis, irrespective of the type of cannabis that was used [44]. Compton et al. (2009) found that past cannabis use had no significant effect on the risk of the onset of psychosis. However, daily cannabis use did increase the risk of the onset of psychosis (hazard ratio (HR) = 1.997, *P* < 0.05) compared to non-cannabis use [45]. According to a 2019 study by Karcher et al., individuals who reported frequent lifetime cannabis use (defined as cannabis use >100 times lifetime) were diagnosed with a cannabis use disorder (CUD) and those who reported current cannabis use had an increased risk of 1.21 to 1.26 times of at least one psychotic-like experience. Moreover, psychotic-like experiences were associated with frequent cannabis use (β = 0.11, 95% CI 0.08–0.14), cannabis use disorder (β = 0.13, 95% CI 0.09–0.16), and current cannabis use (β = 0.07, 95% CI 0.04–0.10), even after adjustment for covariates (*P* < 0.05) [46]. Leadbeater and colleagues (2019) reported that more frequent cannabis use (β = 0.13, 95% CI 0.002–0.25, *P* < 0.05), as well as CUD (β = 0.51, 95% CI 0.01–1.01, *P* < 0.05), during adolescence were associated with psychotic symptoms at ages 22 and 23 [47]. Finally, Marconi and colleagues (2016) performed a meta-analysis of 18 studies reporting on the level of cannabis use that were published before 2014 and demonstrated that higher levels of cannabis use were associated with an increased risk for psychosis in all the included studies. A logistic regression model gave an OR of 3.90 (95% CI 2.84–5.34) for the risk of psychotic disorder among the heaviest cannabis users compared to the non-users [27]. 

In summary, all studies published in the last decade that investigated patterns of cannabis use indicate that frequent cannabis use, in particular daily use, is associated with an increased risk for both psychotic-like experiences and psychotic disorders.

#### 3.1.2. Age of Onset of Cannabis Use

In the past decade, six studies have been published that have investigated the influence of age of initiation of cannabis use on the development of psychosis. Using an online version of the Community Assessment of Psychic Experiences (CAPE) questionnaire in a group of more than 17,000 adolescents, Schubart and colleagues (2011) showed that cannabis use at age 12 or younger was associated with an OR of 3.1 (95% CI 2.1–4.3, *P* < 0.05) for a top 10% score on the subscale measuring psychotic experiences. This significant association between cannabis use and the presence of psychotic experiences was absent when cannabis use was initiated at 15 years or older [48]. Van Gastel and colleagues (2012) found similar effects in a survey of secondary school children aged 12–16 (*N* = 4552), showing that an earlier age of initiation of heavy cannabis use was significantly related to the onset of psychotic experiences (β = 0.065, *P* < 0.001) [49]. In a cohort study of 1756 adolescents, Gage et al. (2014) investigated cannabis use at age 16 in relation to the emergence of psychotic experiences at age 18 and found that frequent cannabis use increased the odds of psychotic experiences (OR = 1.48, 95% CI 1.18–1.86, *P* = 0.001). Nevertheless, controlling for tobacco or other drugs attenuated the odds to 1.2 (95% CI 0.91–1.76, *P* = 0.160) and 1.25 (95% CI 0.91–1.73, *P* = 0.165), respectively, indicating that the use of other substances may be involved in the association [50]. In a cohort of 410 FEP patients, Di Forti et al. (2014) demonstrated that those who had started cannabis at age 15 or younger had an earlier onset of psychosis than those who had started after 15 years (27.0 ± 6.2 vs. 29.1 ± 8.5; HR = 1.40; 95% CI 1.06–1.84, *P* = 0.05) [51]. In the 2019 study by Di Forti and colleagues, they found that individuals who had started using cannabis when 15 years old or younger presented increased odds of psychosis (crude OR = 3.9, 95% CI 3.0–4.9, *P* < 0.0001) [44]. Finally, Setién-Suero and colleagues (2018) showed a significant correlation between age of cannabis use onset and age of onset of psychosis in FEP patients (rho = 0.441; *P* ≤ 0.001). However, there were no significant differences in the age of psychosis onset between early and late cannabis users [52]. 

Overall, the above-mentioned studies indicate that the early initiation of cannabis use is related to an increased risk of developing psychosis. However, because only a limited number of studies have compared the odds of psychosis between early and late cannabis users, it is still unclear if young cannabis users are at a higher risk than late cannabis users. Confounding factors, such as the use of other substances or the cumulative amount of consumed cannabis, should be taken into account as these may explain part of the risk. 

#### 3.1.3. Type of Cannabis Used

Four studies have reported on the type of cannabis used in relation to the risk of psychosis. First, Di Forti and colleagues reported, in their study from 2009, that 78% of the cannabis-using FEP patients consumed high-potency cannabis (containing 12–18% THC) compared with 37% of the control group (OR = 6.8, 95% CI 2.6–25.4, *P* < 0.05) [22]. In a 2015 follow-up study by Di Forti et al., it was reported that the use of high-potency cannabis (containing >10% THC) increased the odds of a psychotic disorder to almost 3.0 compared to those who never used cannabis (OR = 2.92, 95% CI 1.52–3.45, *P* = 0.001). Importantly, everyday use of high-potency cannabis further increased the risk of psychotic disorder (OR = 5.4, 95% CI 2.81–11.31, *P* = 0.002) [53]. In their 2019 study, Di Forti and colleagues concluded that the use of high-potency cannabis (containing >10% THC) modestly increased the odds of a psychotic disorder (OR = 1.5, 95% CI 1.1–2.6), and correction for daily use did not change this. However, the daily use of high-potency cannabis did substantially increase the odds of psychotic disorder (OR = 4.8, 95% CI 2.5–6.3). There was no evidence of an interaction between the frequency of use and type of cannabis used [44]. Finally, based on the results from their 2011 online survey, Schubart et al. reported that the use of cannabis with a low THC:CBD ratio (<55) was related to fewer psychotic experiences than cannabis with a high THC:CBD ratio (>55) (F(1,1877):14.577; *P* < 0.001) [54]. 

In summary, these results suggest that the use of high-potency cannabis (i.e., cannabis with a high THC content or a high THC:CBD ratio) significantly increases the risk of a psychotic disorder.

#### 3.1.4. Genetics

**AKT1 Gene**

Three studies have investigated the moderating effect of the A*KT1* genotype (rs2494732 locus) on the association between cannabis use and the risk of psychosis. AKT1 is a protein kinase involved in the dopamine signaling cascade downstream of the D2 dopamine receptor [55]. Van Winkel et al. (2011) investigated individuals affected by psychosis and their unaffected siblings, and showed that carriers of the C/C genotype had an approximately two-fold increased odds of being diagnosed with a psychotic disorder when using cannabis (OR = 2.08, 95% CI 0.92–4.67) [56]. Di Forti and colleagues found, in a 2012 study, that daily cannabis use of C/C genotype carriers severely increased the odds of a psychotic disorder (OR= 7.23, 95% CI 1.37–38.12) compared to T/T carriers [57]. Finally, Morgan and colleagues (2016) also showed an association between the *AKT1* genotype and cannabis use in the emergence of positive psychotomimetic symptoms, which increased with the C/T or C/C genotype (β = 0.119, *P* = 0.0015) [58].

**COMT Gene**

Nine studies have investigated the influence of the *COMT* Val158Met genotype (rs4680 locus) on the relationship between cannabis use and the incidence of psychosis. Variation in the *COMT* Val158Met polymorphism is associated with dopamine turnover in the prefrontal cortex, with Val/Val carriers having higher COMT enzyme activity and thus reduced dopamine levels compared to Met/Met carriers [59,60]. Henquet and colleagues found, in 2009, that individuals with the Val allele showed an increase in hallucinations after cannabis exposure compared to Met allele carriers [61]. The interaction between *COMT* and cannabis use (F = 3.556; *P* = 0.007; Eta Squared (η^2^)= 0.044) had a significant effect on the age of psychosis onset in a 2010 study by Pelayo-Terán (F = 3.816; *P* = 0.024; η^2^ = 0.045) [62]. Costas and colleagues (2011) demonstrated a doubled probability of lifetime cannabis use in individuals homozygous for the Met allele compared to the homozygous Val genotype (Mantel–Haenszel OR = 2.07, 95% CI 1.27–3.26). There was no significant difference between the different genotypes [63]. Nieman and colleagues (2016) reported an interaction effect between the *COMT* Val158Met genotype and cannabis use on subclinical psychotic symptoms in subjects at CHR for psychosis [64]. 

In contrast, cannabis consumption was not associated with the *COMT* Val158Met polymorphism according to Gutiérrez and colleagues (2009). However, for female regular cannabis users, the psychosis risk was highest when they were also homozygous carriers of the Val allele. Importantly, this seemingly dose-dependent interaction between cannabis consumption, risk for psychosis, and carrying the Val allele in females was not significant [65]. Similarly, Zammit et al. concluded from their 2011 study that there was no evidence of an interaction between cannabis use and six *COMT* single nucleotide polymorphisms (SNPs) in the risk of developing psychotic symptoms [66]. In accordance with these findings, a 2015 study on the rs4680 SNP in a Pakistani population concluded that there was no association between cannabis use and the polymorphism in schizophrenia [67]. Mané and colleagues (2017) studied the interaction between *COMT* and cannabis use with respect to FEP. They reported that early cannabis use and the presence of the *COMT* Val158Met polymorphism were not significantly associated with an earlier onset of psychosis [68]. Another study published in 2017 by Lodhi and colleagues failed to demonstrate a significant moderating effect of the COMT genotype on the age of psychosis onset in cannabis users that initiated use before 20 years of age (*P* = 0.051) [69]. 

**NOS1AP, DRD2, BNDF, and FAAH**

In the last decade, four other genes related to cannabis use and psychosis have been examined, namely, *NOS1AP*, *DRD2*, *BDNF*, and *FAAH*. First, Husted et al. (2012) demonstrated that the presence of the *NOS1AP* risk of the schizophrenia genotype did not influence schizophrenia expression associated with cannabis use [70]. Colizzi and colleagues (2015) investigated the dopamine receptor *D2* (*DRD2*) genotype rs1076560 locus and found that cannabis-using carriers of the T-allele had a three-fold increase in the odds of psychosis compared to GG carriers (OR = 3.07; 95% CI 1.22–7.63). Among daily cannabis users, T carriers showed a five-fold increased odds of psychosis (OR = 4.82; 95% CI 1.39–16.71) [71]. The presence of the Met allele on the brain-derived neurotrophic factor (*BDNF*) gene Val66Met polymorphism was not associated with early cannabis use according to a 2017 study by Mané et al. [68]. Bioque et al. (2019) found that T/T carriers of the Fatty Acid Amide Hydrolase (*FAAH)* rs2295633 SNP (encoding the FAAH enzyme which is involved in the reuptake and degradation of endogenous cannabinoid ligands) had a ten-fold increased probability of presenting with FEP (OR = 10.36, statistical power 0.78), if they used cannabis frequently [72].

**Mendelian Randomization Studies**

Mendelian randomization studies use genetic variants or polygenic scores as instrumental variables to control for gene–environment correlation while estimating the association between an exposure and outcome. In the last decade, three Mendelian randomization studies have been performed that have focused on the potential causal relationship between cannabis use and psychosis. First, Gage and colleagues (2017) found some evidence consistent with a causal effect of cannabis initiation on the risk of psychosis (OR = 1.04, 95% CI 1.01–1.07). However, there was strong evidence consistent with a causal effect of psychosis risk on the likelihood of cannabis initiation (OR = 1.10, 95% CI 1.05–1.14) [73]. A second study performed in 2018 by Vaucher et al. demonstrated that the use of cannabis was associated with an increased risk of psychosis (OR for users vs. nonusers of cannabis = 1.37, 95% CI 1.09–1.67), thereby supporting epidemiological studies arguing that the use of cannabis is causally related to psychosis risk [74]. Finally, Pasman and colleagues (2018) replicated the findings of Gage et al. by showing some weak (non-significant) evidence for a causal influence of lifetime cannabis use on psychosis risk (OR = 1.10, 95% CI 0.99–1.21, *P* = 0.074). They also found stronger evidence for a causal positive influence of psychosis risk on lifetime cannabis use (OR = 1.16, 95% CI 1.06–1.27, *P* = 0.001) [75]. Overall, Mendelian randomization studies suggest that the association between cannabis and psychosis may be bidirectional. 

In summary, evidence exists for a moderating effect of the A*KT1* genotype (rs2494732 locus) on the association between cannabis use and the risk of psychosis, with the highest risk for C/C carriers. Several studies have shown the COMT Val158Met genotype to be a moderator of the relationship between cannabis use and the later development of a psychotic disorder, with the highest risk for Val carriers. However, a significant amount of studies have failed to show an impact on the association between cannabis and psychosis. Interestingly, both dopamine receptor *D2* and FAAH genotypes appear to modulate the effect of cannabis use on the development of psychosis, but these initial findings need to be replicated. Using genetic approaches, Mendelian randomization studies suggest that the association between cannabis and psychosis may be bidirectional. 

### 3.2. Cannabis Use and the Age of Onset of Psychosis

Twenty-two studies have reported on the association between cannabis use and the age of onset of psychosis. In a 2009 study, lifetime cannabis abuse or dependence was associated with a significantly earlier onset of psychosis (β = −3.11, *t*(198) = −3.54; *P* < 0.001) by 3.1 years (95% CI = 1.4–4.8 years earlier). The age at onset was another 3 years earlier in individuals with lifetime cannabis abuse or dependence [76]. Furthermore, progression to daily cannabis use over time predicted an earlier onset of illness or prodromal symptoms (HR = 2.065, *P* < 0.05) [45]. In a 2010 study by Barrigón and colleagues, the age of onset of psychosis treatment was significantly associated with cannabis use; the earlier the age of onset of the heaviest use, the earlier the start of treatment, indicating a dose-response relationship (sex-adjusted log-rank χ^2^(1) = 23.43, *P* < 0.001) [77]. First-episode schizophrenia patients with CUD seemed to have an earlier onset of psychotic symptoms according to Sevy et al. (2010). This result did not hold in a multivariate analysis when additional variables related to CUD were taken into account [78]. In contrast, Schimmelmann and colleagues showed, in 2011, that CUD subjects did not have an earlier age of onset than non-CUD subjects (F(1) = 3.4; *P* = 0.067; η_p_^2^ = 0.01). However, early CUD (starting at age 14 or younger) was associated with an earlier onset of psychosis (β = −0.49, R^2^-change = 0.25, *P* < 0.001) [79]. A systematic meta-analysis by Large and colleagues published in 2011 reported that cannabis use was associated with an earlier onset of psychosis, with the onset for cannabis users being 2.7 years earlier (32 months) (Standardized Mean Difference (SMD) = −0.414, effect size −2.70 years) [80]. Accordingly, cannabis use predicted an earlier age at onset (1.5 years) in schizophrenia patients [81], and cannabis users had a 1.5-year earlier onset of psychosis compared to non-drug users, as reported in 2012 by Dekker and colleagues (difference = 1.7, B = −1.7, Standard Error (SE) = 0.6, *t* = −2.6, *P* = 0.009) [82]. Furthermore, as reported by Estrada and colleagues (2011), there was a significant positive relationship between the age at first use of cannabis and the age of the onset of psychosis (β = 1.66, SE = 0.78, *P* = 0.04). They also investigated the role of the *COMT* Val158Met genotype in this association and found an interaction with cannabis use, showing an earlier psychosis onset for Val/Val carriers than for Val/Met and Met/Met carriers (β = −2.72, SE = 1.30, *P* = 0.04) [83]. Grech and colleagues (2012) found that the age at admission, as a proxy measure for the age of onset of psychosis, was significantly different for individuals who tested positive for cannabis in a urine test (age = 24.63) and negative testers (age = 44.63) (Mann–Whitney *P* = 0.001) [84]. Leeson et al. (2012) found that cannabis users had a significantly younger age at onset of psychosis than non-users (F(1,98) = 9.43, *P* = 0.003) [85]. Furthermore, in a 2012 study by Galvez-Buccollini, a significant interaction between age at the initiation of cannabis use and age of onset of psychosis was found (β = 0.4, 95% CI 0.1–0.7, *P* = 0.008) [86]. Allegri and colleagues demonstrated, in 2013, that users of only cannabis experienced psychotic symptoms 6.2 years earlier than individuals who did not use any drugs (24.2 ± 5.0 vs. 32.9 ± 9.8 years; *t*(1) = 4.26; *P* < 0.001) [87]. In addition, in subjects with schizophrenia spectrum disorder (SSD), age at the initiation of cannabis use and age at the onset of psychosis were significantly linearly associated after adjusting for confounders (F(11,984) = 13.77, *P* < 0.001) [88]. Similarly, after adjustment for diagnosis and gender, FEP patients who used cannabis had an earlier onset of psychosis than abstinent patients (F(1,291) = 16.29, *P*< 0.001) [89]. Di Forti and team (2014) also showed that daily users of high-potency cannabis had an earlier onset of psychosis compared to non-cannabis users of 6 years on average (HR = 1.99; 95% CI 1.50–2.65; *P* < 0.0001) [51]. Additionally, in a 2014 study by Donoghue and colleagues, use of cannabis before the onset of psychosis was associated with an earlier onset of symptoms. In the same study, it was shown that cannabis use interacting with gender was the most parsimonious model influencing the age of onset of symptoms [90]. Among 555 FEP patients, history of cannabis abuse was associated with a nearly 6-year earlier onset of psychosis in 2015 (22.8 vs. 28.7 years, Z = −5.9 years, *P* < 0.001) [91]. Finally, cannabis use was significantly associated with a 3-year earlier onset of SSD in a large multi-site sample (*N* = 1119) study published in 2016, while other factors did not influence the age of onset (F(1,1003) = 31.66, *P* < 0.001) [92]. 

In contrast to the above-mentioned findings, one study reported, in 2009, that the use of cannabis prior to the age of 18 was not associated with cannabis use in individuals with psychotic disorders [93]. DeRosse and colleagues (2010) also concluded that schizophrenia patients with CUD did not have an earlier onset compared to patients without CUD [94]. Finally, in a sample of 633 schizophrenia patients that was subdivided based on the presence of large, rare genetic deletions or large, rare duplications, only those without large duplications had an earlier age of onset related to cannabis abuse [95].

In summary, the vast majority of studies published in the last decade investigating the age of onset of psychosis in relation to cannabis use have found that cannabis use is related to an earlier onset of psychotic symptoms, psychotic experiences, or a psychotic disorder. A few studies have shown an association between an earlier onset of cannabis use and earlier onset of psychosis. 

### 3.3. Cannabis Use and the Transition to Psychosis in CHR Subjects

Five studies have examined cannabis use and the transition to psychosis in CHR individuals. In a study published in 2012, lifetime cannabis abuse was not shown to be related to the transition to psychosis in a small sample of people at CHR for psychosis (*N* = 15) [96]. In a much bigger sample of 182 CHR individuals, Valmaggia and colleagues (2014) investigated the influence of cannabis use on the transition to psychosis and reported that transition was more prevalent in frequent compared to non-frequent users (χ^2^(1) = 4.994, *P* = 0.025). Furthermore, the prevalence was higher in early-onset users (i.e., <age 15) who continued to use frequently compared to late-users (χ^2^(1) = 7.093, *P* = 0.008). However, in the overall sample, transition rates were not significantly increased in cannabis users compared to non-users (χ^2^ (1) = 1.061, *P* = 0.303) [97]. A study by Auther et al. (2015) reported a more prevalent and earlier transition in CHR individuals diagnosed with cannabis abuse or CUD compared to non-users (log-rank test − χ^2^ = 4.67, *P* = 0.031) and cannabis users without impairment (log-rank test − χ^2^ = 4.92, *P* = 0.027). However, adjusting for alcohol use weakened this relationship (HR = 1.875, CI 0.963–3.651, *P* = 0.064), which suggests that the association between cannabis misuse and the transition to psychosis was confounded by alcohol use [98]. McHugh and colleagues demonstrated, in 2017, that individuals with a history of cannabis-induced attenuated psychotic symptoms were 4.90 (95% CI 1.93–12.44) times more likely to develop a psychotic disorder. In these individuals, greater cannabis abuse also indicated a greater risk of transition [99]. Finally, a meta-analysis of seven studies published before 2016 by Kraan et al. (2017) concluded that current cannabis abuse or dependence in subjects at CHR for psychosis increased the odds of transition to 1.75 (95% CI 1.135–2.710) [100].

Taken together, these studies indicate that frequent cannabis use, lifetime cannabis abuse, or cannabis dependence increases the risk of the transition to psychosis in subjects at CHR. However, this effect may be most pronounced in heavy cannabis users and may be confounded by the use of alcohol or other drugs of abuse.

## 4. Discussion

The current systematic review presents a detailed and up-to-date literature overview on factors that influence the relationship between cannabis use and psychosis risk. Overall, the results show that, in particular, frequent cannabis use, especially daily use, and the consumption of high-potency cannabis (i.e., cannabis with high THC and low CBD concentrations) are associated with a higher risk of developing psychosis. Several genotypes have been shown to moderate the impact of cannabis use on psychosis risk, particularly those involved in the dopamine function, such as *AKT1*. Finally, the results indicate that cannabis use is associated with an earlier onset of psychosis and increased risk of transition in individuals at CHR of psychosis. 

High cannabis exposure (i.e., more than weekly, especially daily use) and the use of high-potency cannabis are factors particularly associated with an increased risk of developing psychosis. Although more research is needed to clarify the specific effect of cannabis use in subjects at CHR for psychosis, most studies indicate that cannabis use increases the risk of the transition to psychosis. Since the genetic profile seems to modulate the risk cannabis use poses for the development of psychosis, genetic predisposition should be taken into account when exploring the risk of developing psychotic symptoms of illness. A better integration and understanding of genetic and environmental factors is needed in order to identify the individuals that are most sensitive or more resilient to the effects of cannabis on the risk of psychosis. Future studies should focus on developing methods to identify those individuals with either resilient or vulnerable genotypes, while considering cannabis use within the whole exposome [101]. In addition, the biological mechanisms underlying the influence of cannabis on psychosis risk remain largely unclear [3,30]. Study of the endocannabinoid system might help to find new markers of illness and recovery, and identify new routes for the development of novel treatments [30]. Importantly, CBD, a non-intoxicating compound of cannabis, has shown promising effects in individuals with psychotic symptoms [29,102,103]. Future studies could assess the effect of cannabis with a high CBD content on psychotic symptoms. In any case, there is enough evidence to set up educational programs to inform cannabis users about the risks for developing psychosis, especially in those with a family history of psychosis or at CHR.

When studying single candidate genes, *AKT1* and *COMT* have frequently been associated with cannabis use and an increased risk of psychosis. All recent studies that have investigated the moderating effect of the *AKT1* genotype on the relationship between cannabis and psychosis have concluded that C carriers of the rs2494732 SNP have an increased risk of developing psychosis after cannabis use [56,57,58]. Interestingly, *AKT1* C/C carriers showed an increased sensitivity to the psychotic effects of THC and to THC-induced memory-related activity in both the striatum and midbrain, which further suggests that psychotic effects of cannabis might be mediated by dopamine [104]. Regarding the *COMT* gene, several studies have shown the Val158Met genotype to be a moderator of the association between cannabis use and the later development of a psychotic disorder, with the highest risk for Val carriers [61,62,63,64]. However, a significant number of studies have failed to show an impact on the relationship between cannabis and psychosis [65,66,67,68,69]. As was also concluded by a recent meta-analysis [105], an association between the *COMT* Val158Met genotype, cannabis use, and psychosis cannot be proven at the moment, accounting for the dissimilarity in results. These controversial results probably reflect the limited value of the study of single genes for predicting mental health outcomes [106,107], since complex phenotypes like psychosis are associated with multiple genes [108]. Over the past decade, advanced genetic analyses that allow complex phenotypes to be predicted have been developed, such as polygenic risk scores [109]. Future studies should investigate the moderating effect of psychosis polygenic risk scores on the association between cannabis use and the development of psychosis, rather than examining single candidate genes. Mendelian randomization studies that use genetic variants or polygenic scores could shed further light on causality and the direction of the relationship between cannabis and psychosis. 

A few factors should be taken into account when interpreting the results from the present systematic review. First, the influence of childhood trauma, the use of substances other than cannabis, and exposure to other environmental factors on the relationship between cannabis use and psychotic disorders was beyond the scope of the current review. However, several studies have demonstrated an impact of childhood trauma on the association between cannabis use and psychosis risk [110,111,112]. Additionally, cannabis use is related to the use of other substances (e.g., tobacco) that have independently been associated with psychosis [113], which may have an effect on the relationship between cannabis and psychosis. Therefore, future longitudinal studies may attempt to integrate these potential moderators when studying this relationship. Second, definitions of cannabis use parameters differed between studies. For example, frequent cannabis use was defined as weekly use in some studies, but as daily use in others. Likewise, the definition of high-potency cannabis varied between studies, and actual THC/CBD concentrations of cannabis samples were not assessed. Standardized measures for assessing cannabis use and determining THC/CBD concentrations in cannabis are highly needed for improving the comparison and interpretation of results [114,115]. Third, although the present systematic review provides an up-to-date and detailed overview of the most recent literature, possible differences in the quality of the included studies were not assessed in a systematic way. Therefore, variation in, for example, the study design and sample sizes, ranging from international multicenter studies with standardized assessments of thousands of patients to retrospective measures in smaller patient groups, may explain the differences in reported findings. Finally, the present review did not necessarily focus on confounders of the relationship between cannabis use and psychosis. A few studies have shown that confounding factors, such as age, gender, and the use of other substances, may attenuate the effect of cannabis use on the risk of psychosis [50]. Future studies or literature reviews could clarify which factors are important and could give more insight into the precise effect of cannabis use on the incidence of psychosis. 

In conclusion, literature from the last decade shows that, in particular, frequent cannabis use and the consumption of high-potency cannabis increase the risk of psychosis. Furthermore, cannabis use lowers the age of onset of psychosis by 3 years, and increases the risk of transition in subjects at CHR for psychosis. Cannabis use is a risk factor—not necessary or sufficient for causing psychosis—that interplays with the genetic background, together with other environmental factors. These gene–environmental interactions are complex and still unclear. Further research should aim to clarify which genes are implicated in modulating the effect of modifiable environmental factors like cannabis use within the whole exposome. This might help to predict how changes in these environmental factors can positively impact the prognosis of patients with psychosis or at CHR by counterbalancing a vulnerable genetic profile. In addition, new studies should focus on the biological effect of cannabis on the endocannabinoid system and its relation with psychosis, as well as the potential pharmaceutical properties of CBD. 

## Figures and Tables

**Table 1:** Explanation of statistical outcome measures.

**OR**	Odds ratio	An OR is a statistic that quantifies the strength of the association between two events, for example, the use of cannabis and the development of a psychotic disorder. An OR greater than 1 indicates that the two events are associated.
**HR**	Hazard ratio	HR is a measure of an effect of an intervention on an outcome of interest over time, for example, daily cannabis use and the onset of psychosis. An HR of 1 indicates that event rates (e.g., onset of psychosis) are the same in both groups (e.g., daily cannabis use vs. no cannabis use).
**Regression beta coefficient**		A regression assesses whether predictor variables (e.g., age of onset of cannabis use) account for variability in a dependent variable (onset of psychosis). The beta coefficient is the degree of change in the outcome variable for every 1-unit of change in the predictor variable. If the beta coefficient is positive and significant, the interpretation is that for every 1-unit increase in the predictor variable, the outcome variable will increase by the beta coefficient value.

## Supplementary Materials

The following are available online at https://www.mdpi.com/2076-3425/10/2/97/s1, Table S1: Overview of studies included in the present systematic review.
