# Supplementary material for: Factors Moderating the Association between Cannabis Use and Psychosis Risk: A Systematic Review
Source: Brain Sci. 2020 Feb 12;10(2):97. doi: 10.3390/brainsci10020097 (PMC7071602; doi:10.3390/brainsci10020097)
Supplement: Supplementary file 1 [file brainsci-10-00097-s001.pdf]

Systematic Review

Factors Moderating the Association Between Cannabis Use and Psychosis Risk: A Systematic Review

Sanne J. van der Steur [1], Albert Batalla [1], Matthijs G. Bossong [1*]

[1] Department of Psychiatry, University Medical Center Utrecht Brain Center, Utrecht University, Utrecht, the Netherlands

* Correspondence: m.bossong@umcutrecht.nl; Tel.: +31-88-7556369

Supplementary Materials

Table 1. Overview of studies included in the present systematic review

Authors and year	Title	Journal	Subheading (nr)
Allegri et al. (2013)	Current cannabis use and age of psychosis onset: A gender-mediated relationship? Results from an 8-year FEP incidence study in Bologna.	*Psychiatry Research*, *210*(1), 368–370	3.2 AOP
Auther et al. (2015)	Alcohol confounds relationship between cannabis misuse and psychosis conversion in a high-risk sample.	*Acta Psychiatrica Scandinavica*, *132*(1), 60–68	3.3 Transition in CHR subjects
Auther et al. (2012)	Prospective Study of Cannabis Use in Adolescents at Clinical High Risk for Psychosis: Impact on Conversion to Psychosis and Functional Outcome.	*Psychological medicine* 42(12): 2485–97	3.1 Type of cannabis
Barrigón et al. (2010)	Temporal Relationship of First-Episode Non-Affective Psychosis with Cannabis Use: A Clinical Verification of an Epidemiological Hypothesis.	*Journal of Psychiatric Research*, *44*(7), 413–420	3.1 Genetics
Bioque et al. (2019)	Gene-Environment Interaction between an Endocannabinoid System Genetic Polymorphism and Cannabis Use in First Episode of Psychosis.	*European Neuropsychopharmacology* 29(6): 786–94	3.1 Patterns of cannabis use
Colizzi et al. (2015)	Interaction between Functional Genetic Variation of DRD2 and Cannabis Use on Risk of Psychosis.	*Schizophrenia Bulletin, 41(5), 1171–1182*	3.1 Genetics
Compton et al. (2009)	Association of Pre-Onset Cannabis, Alcohol, and Tobacco Use with Age at Onset of Prodrome and Age at Onset of Psychosis in First-Episode Patients.	*American Journal of Psychiatry, 166(11), 1251–1257*	3.2 AOP
Costas et al. (2011)	Interaction between *COMT* haplotypes and cannabis in schizophrenia: A case-only study in two samples from Spain.	*Schizophrenia Research*, *127*(1–3), 22–27	3.1 Genetics

Dekker et al. (2012)	Age at Onset of Non-Affective Psychosis in Relation to Cannabis Use, Other Drug Use and Gender	*Psychological Medicine, 42(9), 1903–1911*	3.2 AOP
DeRosse et al. (2010)	Cannabis Use Disorders in Schizophrenia: Effects on Cognition and Symptoms.	*Schizophrenia Research* 120(1–3): 95–100	3.2 AOP
Di Forti et al. (2012)	Confirmation that the *AKT1* (rs2494732) genotype influences the risk of psychosis in cannabis users.	*Biological Psychiatry*, *72*(10), 811–816	3.1 Genetics
Di Forti et al. (2015)	Proportion of patients in south London with first-episode psychosis attributable to use of high potency cannabis: A case-control study.	*The Lancet Psychiatry*, *2*(3), 233–238	3.1 Type of cannabis
Di Forti et al. (2009)	High-potency cannabis and the risk of psychosis.	*British Journal of Psychiatry*, *195*(6), 488–491	3.1 Type of cannabis
Di Forti et al. (2019)	The contribution of cannabis use to variation in the incidence of psychotic disorder across Europe (EU-GEI): a multicentre case-control study.	*The Lancet Psychiatry, 6(5), 427–436*	3.1 Patterns + type of cannabis use
Di Forti et al. (2014)	Daily Use, Especially of High-Potency Cannabis, Drives the Earlier Onset of Psychosis in Cannabis Users.	*Schizophrenia Bulletin*, 40(6), 1509–1517	3.1 Age of onset of cannabis use, 3.2 AOP
Donoghue et al. (2014)	Cannabis use, gender and age of onset of schizophrenia: Data from the ÆSOP study.	*Psychiatry Research*, *215*(3), 528–532	3.2 AOP
Estrada et al. (2011)	Cannabis Use and Age at Onset of Psychosis: Further Evidence of Interaction with *COMT* Val158Met Polymorphism.	*Acta Psychiatrica Scandinavica, 123*(6), 485–492	3.2 AOP
Gage et al. (2014)	Associations of cannabis and cigarette use with psychotic experiences at age 18: findings from the Avon Longitudinal Study of Parents and Children.	*Psychological Medicine*, *44*(16), 3435–3444	3.2 AOP
Gage et al. (2013)	Using mendelian randomisation to infer causality in depression and anxiety research.	*Depression and Anxiety*, *30*(12), 1185–1193	3.1 Genetics
Galvez-Buccollini et al. (2012)	Association between age at onset of psychosis and age at onset of cannabis use in non-affective psychosis.	*Schizophrenia Research, 139*(1–3), 157–160	3.2 AOP
Grech, Camilleri & Taylor (2012)	Cannabis use and age of admission to a psychiatric unit for first episode of psychosis.	*Malta Medical Journal*, *24*(3), 17–20	3.2 AOP
Gutiérrez et al. (2009)	Variability of the *COMT* gene and modification of the risk of schizophrenia conferred by cannabis consumption.	*Revista de Psiquiatria y Salud Mental*, *2*(2), 89–94	3.1 Genetics

Helle et al. (2016)	Cannabis use is associated with 3 years earlier onset of schizophrenia spectrum disorder in a naturalistic, multi-site sample (N = 1119).	*Schizophrenia Research, 170*(1), 217–221	3.2 AOP
Henquet et al. (2009)	*COMT* Val158Met moderation of cannabis-induced psychosis: A momentary assessment study of "switching on" hallucinations in the flow of daily life.	*Acta Psychiatrica Scandinavica, 119*(2), 156–160	3.1 Genetics
Husted et al. (2012)	Early environmental exposures influence schizophrenia expression even in the presence of strong genetic predisposition.	*Schizophrenia Research*, *137*(1–3), 166–168	3.1 Genetics
Karcher et al. (2019)	Genetic Predisposition vs Individual-Specific Processes in the Association between Psychotic-like Experiences and Cannabis Use.	*JAMA Psychiatry*, *76*(1), 87–94.	3.1 Patterns of cannabis use
Kantrowitz et al. (2009)	Adolescent cannabis use, psychosis and catechol-O-methyltransferase genotype in african Americans and caucasians	*Psychiatric Quarterly*, *80*(4), 213–218	3.1 Genetics
Kraan et al. (2016)	Cannabis use and transition to psychosis in individuals at ultra-high risk: Review and meta-analysis.	*Psychological Medicine*, *46*(4), 673–681	3.3 Transition in CHR subjects
Large et al. (2011)	Cannabis Use and Earlier Onset of Psychosis: A systematic meta-analysis	*Archives of General Psychiatry*, *68*(6), 555	3.2 AOP
Leadbeater et al. (2019)	Age-varying effects of cannabis use frequency and disorder on symptoms of psychosis, depression and anxiety in adolescents and adults.	*Addiction*, *114*(2), 278–293	3.1 Patterns of cannabis use
Leeson et al. (2012)	The effect of cannabis use and cognitive reserve on age at onset and psychosis outcomes in first-episode schizophrenia.	*Schizophrenia Bulletin*, *38*(4), 873–880	3.2 AOP
Lodhi et al. (2017)	Investigation of the *COMT* Val158Met variant association with age of onset of psychosis, adjusting for cannabis use.	*Brain and Behavior*, *7*(11), e00850	3.1 Genetics
Mané et al. (2017)	Cannabis use, *COMT*, BDNF and age at first-episode psychosis.	*Psychiatry Research, 250*, 38–43	3.1 Genetics
Marconi et al. (2016)	Meta-analysis of the Association Between the Level of Cannabis Use and Risk of Psychosis.	*Schizophrenia Bulletin*, *42*(5), 1262–1269	3.1 Patterns of cannabis use
Martin et al. (2014)	Cannabis abuse and age at onset in schizophrenia patients with large, rare copy number variants.	*Schizophrenia Research*, *155*(1–3), 21–25	3.2 AOP
McHugh et al. (2017)	Cannabis-induced attenuated psychotic symptoms: Implications for prognosis in young people at ultra-high risk for psychosis.	*Psychological Medicine*, *47*(4), 616–626	3.3 Transition in CHR subjects
Myles et al. (2016)	Cannabis use in first episode psychosis: Meta-analysis of prevalence, and the time course of initiation and continued use.	*Australian and New Zealand Journal of Psychiatry*, *50*(3), 208–219	3.2 AOP

Nawaz & Siddiqui (2015)	Association of single nucleotide polymorphisms in catechol-omethyltransferase and serine-threonine protein kinase genes in the pakistani schizophrenic population: A study with special emphasis on cannabis and smokeless tobacco.	*CNS and Neurological Disorders - Drug Targets*, *14*(8), 1086–1095.	3.1 Genetics
Nieman et al. (2016)	*COMT* Val158Met genotype and cannabis use in people with an At Risk Mental State for psychosis: Exploring Gene x Environment interactions.	*Schizophrenia Research*, *174*(1–3), 24–28	3.1 Genetics
O'Donoghue et al. (2015)	Environmental factors and the age at onset in first episode psychosis.	*Schizophrenia Research*, *168*(1–2), 106–112	3.2 AOP
Öngür et al. (2009)	Clinical characteristics influencing age at onset in psychotic disorders.	*Comprehensive Psychiatry*, *50*(1), 13–19	3.2 AOP
Pasman et al. (2018)	GWAS of lifetime cannabis use reveals new risk loci, genetic overlap with psychiatric traits, and a causal effect of schizophrenia liability.	*Nature Neuroscience*, *21*(9), 1161–1170	3.1 Genetics
Pelayo-Terán et al. (2010)	Catechol-O-Methyltransferase (*COMT*) Val158Met variations and cannabis use in first-episode non-affective psychosis: Clinical-onset implications.	*Psychiatry Research*, *179*(3), 291–296	3.1 Genetics
Sevy et al. (2010)	Are cannabis use disorders associated with an earlier age at onset of psychosis? A study in first episode schizophrenia.	*Schizophrenia Research*, *120*(1–3), 101–107.	3.2 AOP
Schimmelmann et al. (2011)	Cannabis use disorder and age at onset of psychosis - A study in first-episode patients.	*Schizophrenia Research*, *129*(1), 52–56	3.2 AOP
Schubart et al. (2011)	Cannabis with high cannabidiol content is associated with fewer psychotic experiences.	*Schizophrenia Research*, *130*(1–3), 216–221	3.1 Type of cannabis
Schubart et al. (2011)	Cannabis use at a young age is associated with psychotic experiences.	*Psychological Medicine*, *41*(6), 1301–1310	3.1 Age of onset of cannabis use
Setién-Suero et al. (2018)	Age of onset of Cannabis use and cognitive function in first-episode non-affective psychosis patients: Outcome at three-year follow-up.	*Schizophrenia Research*, *201*, 159–166	3.2 AOP
Stefanis et al. (2013)	Age at initiation of cannabis use predicts age at onset of psychosis: The 7-to 8-year trend.	*Schizophrenia Bulletin*, *39*(2), 251–254	3.2 AOP
Tosato et al. (2013)	The impact of cannabis use on age of onset and clinical characteristics in first-episode psychotic patients. Data from the Psychosis Incident Cohort Outcome Study (PICOS).	*Journal of Psychiatric Research*, *47*(4), 438–444	3.2 AOP
Valmaggia et al. (2014)	Cannabis use and transition to psychosis in people at ultra-high risk.	*Psychological Medicine*, *44*(12), 2503–2512	3.3 Transition in CHR subjects

van Gastel et al. (2012)	Cannabis use and subclinical positive psychotic experiences in early adolescence: findings from a Dutch survey.	*Addiction*, *107*(2), 381–387	3.1 Age of onset of cannabis use
van Winkel et al. (2011)	Family-based analysis of genetic variation underlying psychosis-inducing effects of cannabis: Sibling analysis and proband follow-up.	*Archives of General Psychiatry*, *68*(2), 148–157	3.1 Genetics
Vaucher et al. (2018)	Cannabis use and risk of schizophrenia: a Mendelian randomization study.	*Molecular Psychiatry*, *23*(5), 1287–1292	3.1 Genetics
Vaucher et al. (2017)	Cannabis use and risk of schizophrenia: a Mendelian randomization study.	*Nature Publishing Group*, *23*, 1287–1292	3.1 Genetics
Zammit et al. (2011)	Cannabis, *COMT* and psychotic experiences.	*British Journal of Psychiatry*, *199*(5), 380–385	3.1 Genetics

[1] AOP: age of onset psychosis
